# HIV-1 Infection Is Blocked at an Early Stage in Cells Devoid of Mitochondrial DNA

**DOI:** 10.1371/journal.pone.0078035

**Published:** 2013-10-21

**Authors:** Gaofei Lu, Suzanne E. Matsuura, Antoni Barrientos, Walter A. Scott

**Affiliations:** 1 Department of Biochemistry and Molecular Biology, University of Miami Miller School of Medicine, Miami, Florida, United States of America; 2 Department of Neurology, University of Miami Miller School of Medicine, Miami, Florida, United States of America; George Mason University, United States of America

## Abstract

Human immunodeficiency virus type I (HIV-1) exploits various host cellular pathways for efficient infection. Here we report that the absence of mitochondrial DNA (mtDNA) in ρ^0^ cells markedly attenuates HIV-1 infection. Importantly, reduced infection efficiency in ρ^0^ cells is not simply the result of impaired oxidative phosphorylation (OXPHOS) because pharmacological OXPHOS inhibition did not inhibit HIV-1 infection. Analysis of the early steps of virus infection by real-time PCR quantification of stage-specific HIV-1 DNA products in the infected ρ^0^ and parental cell line have allowed us to conclude that HIV-1 infection in ρ^0^ cells is blocked at the steps that occur after reverse transcription and prior to nuclear import. Additionally, confocal fluorescence microscope analysis showed that the majority of viral complexes containing HIV-1 p24 co-localize with mitochondria in target cells, suggesting an interaction between the two. Collectively, our data strongly indicate that mitochondria play an important role during early stages of HIV-1 infection, probably through direct association with HIV-1 intracellular complexes.

## Introduction

During the early stages of human immunodeficiency virus type I (HIV-1) infection, the viral RNA genome is reverse-transcribed into a double-stranded DNA copy that is subsequently modified by viral integrase and translocated into the nucleus where it is integrated into the host cell chromosome. Various cytoplasmic structures in the target cell have been implicated in these processes, which involves a complex interplay between viral and cellular proteins (reviewed in 1).

These early events in HIV-1 infection are challenging to study because the few incoming viral genomes that give rise to functional integrated proviruses are outnumbered by virus particles that do not complete the full infection cycle. The challenge has been addressed by the use of genetic approaches that depend on functional assays, which have led to the identification of numerous host factors required for productive infection [1-6] and other host factors that restrict HIV-1 infection [7,8]. Host factors have also been identified by biochemical experiments and their biological relevance has been demonstrated by siRNA experiments [9]. These studies have identified numerous host pathways in HIV-1 infection but the molecular mechanisms involved remain in some cases to be fully understood. A case in point is the role or roles of host cell mitochondria during HIV-1 infection.

Most viruses have evolved strategies to prevent viral suppression via apoptosis or exploit mitochondrial pathways to eliminate cells involved in the host immune response [10]. Among them, HIV-1 is known to use anti-apoptotic and apoptotic strategies during infection and acquired immunodeficiency syndrome or AIDS [11,12]. Additionally, AIDS progression in patients is also associated with mitochondrial DNA (mtDNA) depletion [13], disruption of energy production via oxidative phosphorylation (OXPHOS) and increased ROS production [14]. Furthermore, mtDNA depletion in patients is severely aggravated by antiviral drug treatments based on reverse transcriptase inhibitors, which also inhibit the γ-type mtDNA polymerase [15]. However, studies of HIV-1 biology have usually not focused on a role for mitochondria during the early stages of HIV-1 infection. Nonetheless, two of the recent genome-wide siRNA screens in search of proteins required for HIV-1 infection showed unexpected enrichment for genes in pathways associated with mitochondrial function [2,4]. The genome-wide siRNA screen reported by Zhou et al. [4] identified seven mitochondrial proteins required for HIV-1 infection, one of which (TOMM70A) was also identified in the screen reported by Brass et al. [2]. Additional mitochondrial factors (including the F_1_F_O_ -ATP synthase and proteins related to apoptosis) were identified by screens of Brass et al. [2] and Yeung et al. [5]. It is well documented that for viruses other than HIV-1, viral interactions with host cell mitochondrial membranes play critical roles in infection. The best studied involves a host protein, gC1qR/p32, that binds to intracellular nucleoproteins of viruses such as rubella and stimulates virus infection [16-19]. An indirect mechanism for this effect has recently been proposed by Xu et al. [20], who showed that viral infection induces translocation of gC1qR/p32 to the mitochondria where it blocks a mitochondrial intermediate in the innate antiviral response pathway mediated by the RNA helicases RIG1 (retinoic-acid-inducible protein 1) and MDA5 (melanoma differentiation-associated gene 5), which are members of the Rig1-like receptor (RLR) class. In vivo, RIG-I is activated by viral RNA then associates with the mitochondrial antiviral signaling (MAVS) protein to subsequently induce potent inflammatory cytokines to combat the infection [10]. Notably, the localization of MAVS in the outer mitochondrial membrane is essential for its ability to mediate RLR signaling and therefore, the mitochondria act as a platform for antiviral signaling [10]. 

However, despite the high-throughput studies mentioned earlier, a role for mitochondria in mediating productive HIV-1 infection is yet to be described. In this manuscript, we have approached this issue by analyzing HIV-1 infection in cells containing or devoid of mtDNA (ρ^+^ and ρ^0^ cell lines, respectively). We report that the absence of mtDNA in ρ^0^ cells markedly attenuates HIV-1 infection through a mechanism independent of oxidative phosphorylation (OXPHOS). The poor HIV-1 infectivity in ρ^0^ cells stems from a block at an early step of viral infection, after reverse transcription and before nuclear import. Confocal fluorescence microscopy analysis showed a large fraction of HIV-1 particles containing capsid to be near or in contact with mitochondria in the target cells. We conclude that mitochondria play an important role in the early stages of HIV-1 infection, probably through direct association with viral intracellular complexes, thus facilitating intracellular transport of viral complexes.

## Results

### Cells devoid of mtDNA (ρ^0^ cells) do not support HIV-1 infection

To analyze the contribution of mitochondria to the efficiency of HIV-1 infection, we started by characterizing the infectivity of human cells containing or devoid of mtDNA (ρ^+^ and ρ^0^ cell lines, respectively). Human ρ^0^ cell lines were obtained by prolonged passage in medium containing ethidium bromide, which leads to loss of mitochondrial DNA (mtDNA). Conveniently, respiratory null cell lines, including ρ^0^, are able to replicate in culture if the medium is supplemented with pyruvate and uridine [21,22]. Elimination of mtDNA is known to produce functional and morphological changes in mitochondria, which frequently form a fragmented network and have altered structure, with poor development of inner membrane cristae, but has minor effects on nuclear genome and encoded proteins [21,23-28]. The ρ^0^ cell line used here is a previously reported derivative of the human osteosarcoma (HOS) 143B thymidine kinase-deficient (TK^-^) cell line [21]. 

**Figure 1:**
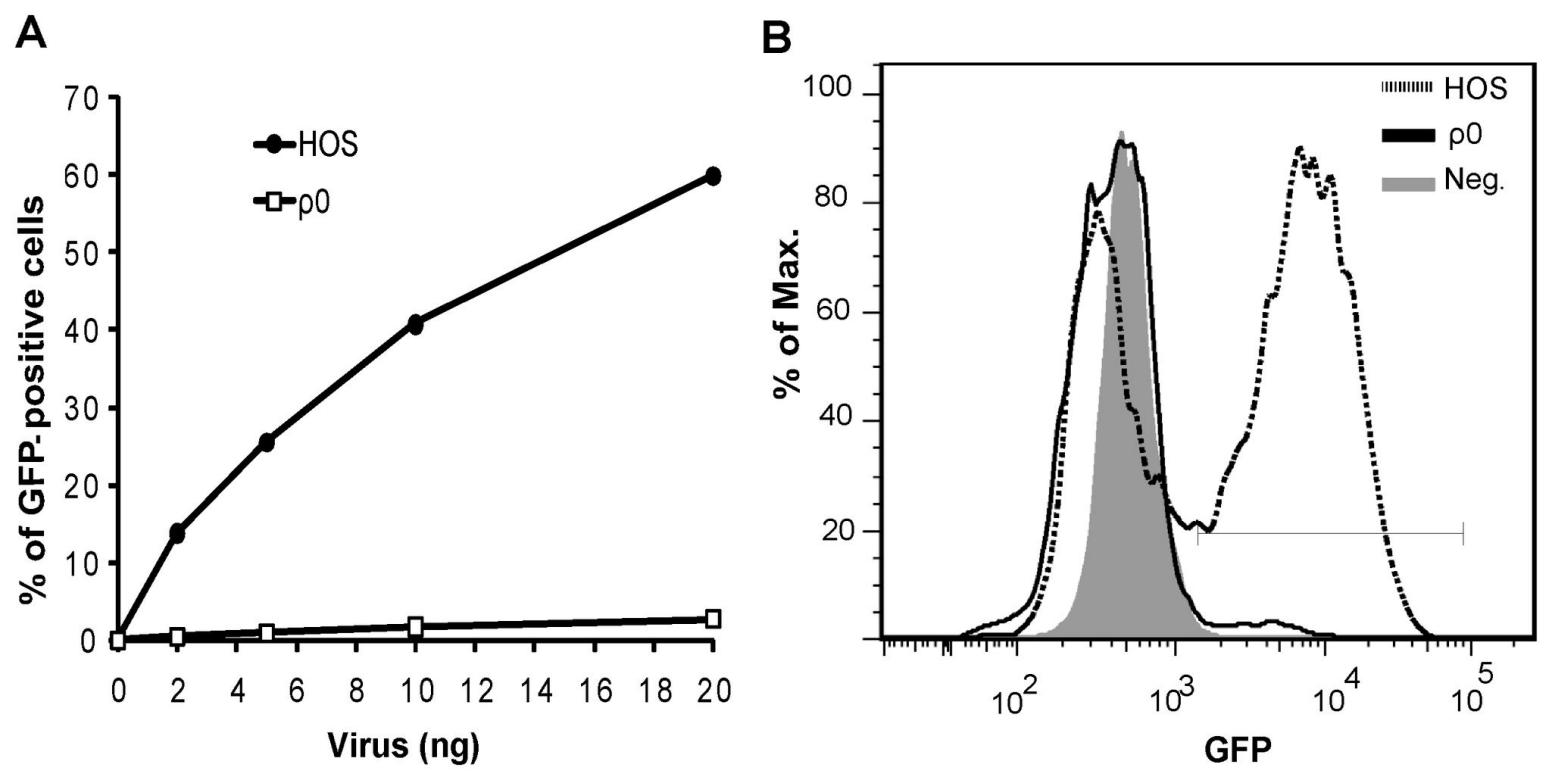
HIV-GFP infection is extremely inefficient in ρ^0^ cells devoid of mtDNA. (*A*) Estimation of cellular infectivity by HIV-1 in HOS and ρ^0^ cells. GFP expression in different cells was measured by FACS analysis and was plotted against the amount of virus used in each experiment. (*B*) Representative histograms of GFP quantification by FACS analysis in cells infected with an equivalent of 20 ng of p24 of HIV-GFP. Gray bar indicates the gate for GFP expression. The graph is representative of three independent experiments.

To test whether mtDNA was necessary for HIV-1 infection we measured the transduction efficiency of a vesicular stomatitis virus – protein G (VSV-G) pseudotyped HIV-1 based lentiviral vector engineered to express green fluorescent protein (HIV-GFP), which is known to retain full infectivity capacity [29]. Virus transduction in ρ^0^ and parental ρ^+^ HOS cells was measured by GFP expression, quantified by FACS analysis, two days post-infection (Figure **1A**). Compared to ρ^+^ HOS cells, an approximately 20-fold decrease in HIV-GFP transduction at virus input equivalent of 20 ng of p24 was observed for ρ^0^ cells (Figure **1B**). This result indicates that the presence of mtDNA is critical for efficient HIV-1 infection, and that some steps during virus infection were blocked in ρ^0^ cells.

### Mitochondrial OXPHOS is not required for efficient HIV-1 infection in HOS cells

MtDNA encodes 13 protein subunits of oxidative phosphorylation (OXPHOS) system enzymes. Therefore, ρ^0^ cells lack an OXPHOS system and are unable to produce aerobic ATP. This supports the hypothesis of a contribution of OXPHOS to HIV-GFP infection. 

**Figure 2:**
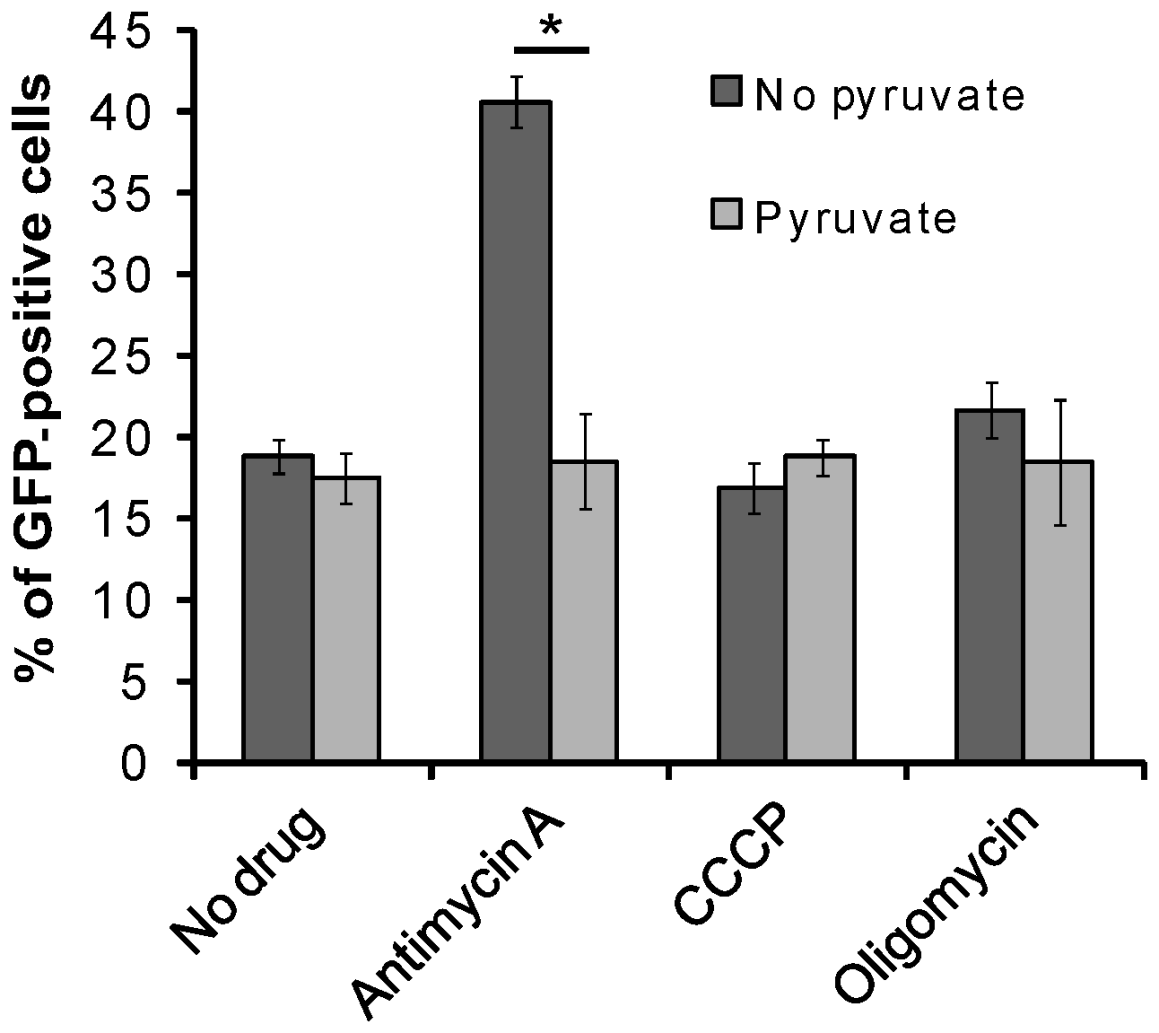
Mitochondrial inhibitors do not lower the efficiency of HIV-GFP infection in HOS cells. HOS cells were pre-treated with the indicated mitochondrial inhibitors for 6 hours and subsequently infected with HIV-GFP virus. Forty-eight hours post-infection, the percentage of GFP expression was measured by FACS analysis. The error bars indicate the standard deviation (n=3). *P<0.001.

To evaluate the contribution of a functional OXPHOS to HIV-GFP infection in ρ^+^ HOS cells we used three mitochondrial poisons to block respiration at the level of complex III (antimycin A), to inhibit the functioning of the F_1_F_O_-ATPase (oligomycin), or to dissipate the proton gradient across the mitochondrial inner membrane, required to drive the synthesis of ATP (the uncoupler CCCP). Routinely, HOS cells were treated with each mitochondrial poison for 6 hours prior to HIV-GFP infection and two days later, virus transduction efficiency was measured by FACS analysis (Figure **2**). The optimal concentrations and effects of these inhibitors on mitochondrial function are well documented [30]. When the growth medium was not supplemented with pyruvate and uridine, cell proliferation was prevented by low concentrations of antimycin A (1.25 µg/ml) as reported [18]. However, growth inhibition by antimycin A could be substantially prevented by the supplementation of pyruvate and uridine, required for growth of OXPHOS null cells (Figure **S1** in File **S1**). In these conditions, the cells remained, however, alive for several days. On the contrary, oligomycin (from 100 ng/ml to 10 μg/ml) and CCCP (up to 10μM), did not inhibit cell growth significantly at any of the concentrations used in this experiment (**not shown**). Abundant cell death was observed by the treatment with high concentrations of CCCP (>20 µM) or oligomycin (20 µg/ml) (**not shown**). As shown in Figure **2**, neither oligomycin nor CCCP treatments altered HIV-GFP infection efficiency. Similarly, HIV-1 transduction efficiency in antimycin A-treated cells (grown in medium supplemented with pyruvate and uridine) was similar to the untreated control cells. Interestingly, the transduction efficiency was doubled in cells treated with antimycin A in medium depleted of pyruvate and uridine, probably due to cell growth inhibition by antimycin A in this medium. The inhibition of cell growth increased the ratio of viral cDNA/cellular genome by 2-fold compared with the cells having normal growth rate (doubling time, 24 hours), which led to 2-fold increase of transduction efficiency. The results presented in this section indicate that inhibition of OXPHOS by mitochondrial inhibitors does not lead to decreased virus infection in HOS cells. 

### The decreased HIV-1 infection efficiency in ρ^0^ HOS cells can be significantly restored by cell repopulation with mtDNA

Human ρ^0^ cells can be repopulated with mtDNA from donor cells such as platelets or fibroblasts from controls and patients with mitochondrial disorders owing to mutations in the mtDNA. The resulting transmitochondrial cybrid cell lines have been extensively used as a model to analyze the cellular phenotypes arising from mtDNA mutations [21,31-33]. 

To further explore the relationship between virus infection and mitochondria, we generated a transmitochondrial cybrid cell line containing functional mtDNA transferred from HEK293T cells, which are thymidine kinase-positive (TK^+^). The use of a TK^+^ cell line as mtDNA donor was required to ensure proper cybrid selection. HEK293T cells converted to cytoplasts by enucleation by actinomycin D treatment were fused to ρ^0^ cells by the polyethylene glycol (PEG)-mediated membrane fusion method [32,33] and clones were selected for their ability to grow in a culture medium lacking pyruvate, pyrimidines and containing bromodeoxyurine (BrdU). Our ρ^0^ cells are auxotrophic for pyruvate and pyrimidines because of the lack of a functional respiratory chain and are TK^-^ [21]. Therefore, only the ρ^0^ cells that had fused with cytoplasts could survive in this medium, while the ρ^0^ cells that had not fused or that had fused with residual intact HEK293T cells, as well as any residual intact HEK293T cells, were eliminated. Selection with BrdU against expression of the TK^+^ phenotype was maintained during the entire culture period. Individual clones were picked after 21 days of selection.

**Figure 3:**
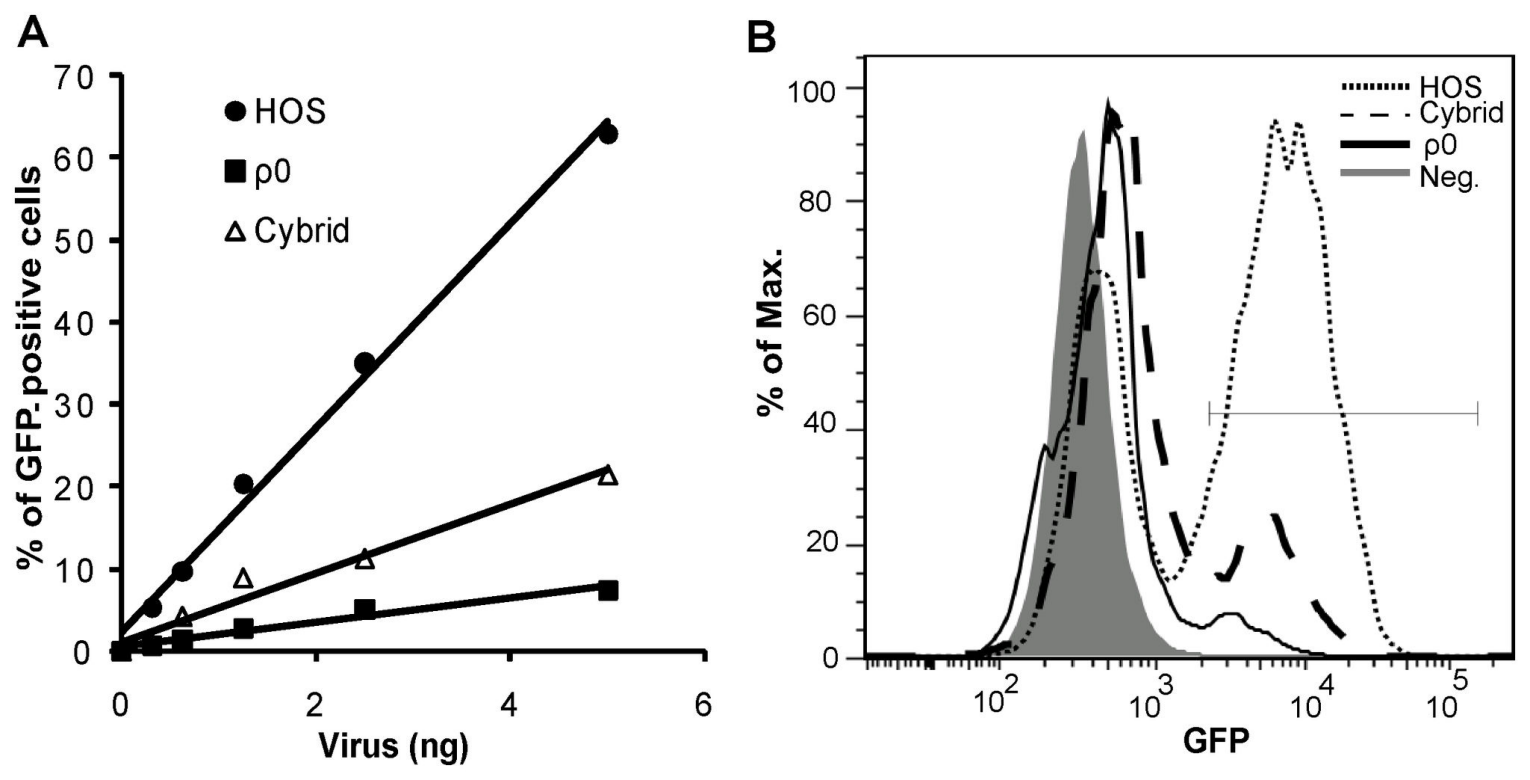
Infection efficiency by HIV-GFP is significantly restored in a transmitochondrial cybrid line. (*A*) Estimation of cellular infectivity by HIV-1 in HOS, cybrid and ρ^0^ cells. HOS, cybrid and ρ^0^ cells were infected with HIV-GFP virus in the presence of 10 µg/ml polybrene for 5 hours and the medium was subsequently replaced with fresh complete medium. Two days later the percentage of GFP expression was measured by FACS analysis and was plotted against the amount of virus used in each experiment. (*B*) Representative histograms of GFP fluorescence quantification by FACS analysis in HOS cells, cybrid, and ρ^0^ cells infected with an equivalent of 5 ng of p24 of HIV-GFP. Grey bar indicates the gate for GFP expression. The graph is representative of three independent experiments.

As a prerequisite for further investigation, we first confirmed by real-time PCR that there was no significant difference in mtDNA levels between the isolated cybrid line and HOS cells (**not shown**). Subsequently, the HIV-GFP transduction efficiency in cybrid cells was tested and compared with that in HOS and ρ^0^ cells (Figure **3**). As shown in Figure **3**, the virus transduction efficiency, which in ρ^0^ cells was 9-fold lower than in HOS cells, was restored by 3-fold in cybrid cells. Although the infection efficiency varied slightly among experiments, these results were confirmed in two independent replicas (Figure **S2** in File **S1**). Full restoration was perhaps prevented by some nuclear/mtDNA incompatibility in cybrid cells resulting from the replacement of HOS mtDNA with HEK293T mtDNA. In this line, it is noteworthy that mtDNA haplogroups [34] as well as genetic variants in nuclear-encoded mitochondrial genes [35] influence AIDS progression. Independently, our results clearly showed that introduction of mtDNA partially but significantly rescued the HIV-GFP infection defect in ρ^0^ cells. 

### HIV-1 infection in ρ^0^ cells is blocked at the steps after reverse transcription and before nuclear import

To explore which aspect of HIV-GFP infection was impaired in the absence of mtDNA, we assessed reverse transcription, nuclear import and DNA integration in the ρ^0^, cybrid and parental ρ^+^ HOS cell lines. For this purpose, we measured by quantitative real-time PCR the levels of stage-specific HIV-GFP DNA products including early reverse transcription (RT) products (strong stop DNA), late RT products (full length cDNA), 2-LTR circles (to analyze nuclear import) and integrated viral DNA. The synthesis of full-length viral cDNA indicates the completion of reverse transcription. HIV-1 2LTR circles, the products of non-homologous end joining DNA repair events, are exclusively found in the nucleus, and serve as a marker of viral nuclear import in studies of viral trafficking. Integration of viral cDNA into the cellular chromosome DNA is the last step of the early stages of virus infection. Virus integration can be measured by the detection of integrated viral DNA by Alu-PCR [36,37].

**Figure 4:**
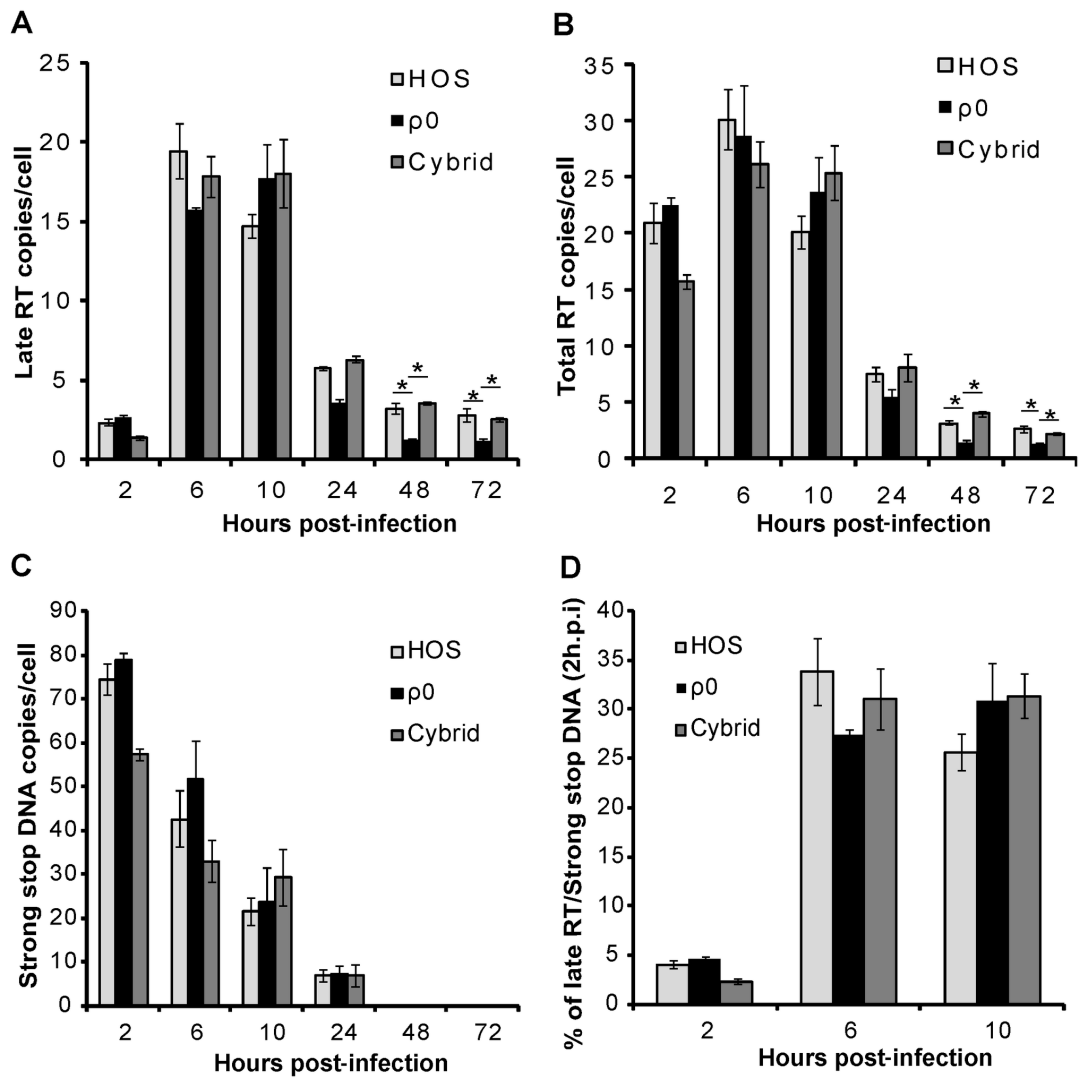
Reverse transcription activity is similar in HOS, cybrid and ρ^0^ cells. Real time PCR quantification of (*A*) HIV-GFP late and (*B*) total RT product, normalized by cellular RNase P in cells infected with DNase I-treated HIV-GFP virus. Values are expressed as viral cDNA copies per cell (RNase P). In this analysis, only (-) single-stranded strong stop DNA and late RT products were considered. One molecule of linearized pWPI (standard) contains two LTR sequences, which is equal to 4 molecules of single-stranded strong stop DNA in a PCR measurement. (*C*) Graph representing single-stranded strong stop DNA values calculated using the formula: *Strong* *stop* *DNA =* (Total RT-Late RT) *X 4*. (*D*) Graph representing the percentage of late RT (2, 6, 10 hours post-infection) per strong stop DNA (2 hours post-infection) to demonstrate the progress and completion of the reverse transcription. The error bars indicate standard deviation of triplicate values. Data are representative of three independent experiments. * *P*<0.01.

For quantitative real-time PCR analyses, we used published primers [36,37] and primers designed specific for the genome sequence of HIV-GFP used in this study (Table **S1** in File **S1**). We considered only early and late RT products because minus strong stop DNA transfer (after the synthesis of strong stop DNA) is a rate-limiting step in reverse transcription. Minus single-stranded strong stop DNA can be calculated by the subtraction of late RT product from total RT product (described in the legend to Figure **4**). Since strong stop DNA (early RT) is the product of first step of reverse transcription and full-length DNA (late RT) is the product of the last step of reverse transcription, comparison of the two permits determination of the progress and completion of the reverse transcription process.

To measure HIV-GFP DNA species, ρ^0^, cybrid and ρ^+^ HOS cell lines were infected with equivalent amounts of DNase I-treated HIV-GFP virus and total cellular DNA was isolated at various post-infection times for qPCR analysis of different viral cDNA products (Figure **4**). In ρ^+^ HOS cells, early RT products (strong stop DNA) were at maximal level, nearly 70 copies per cell at the initial 2 hours and then rapidly decreased (Figure **4C**), and late RT products peaked at around 6~10 hours (nearly 15 copies per cell) and then decreased to approximately 6 copies per cell by 24 hours (Figure **4A**). In cybrid and ρ^0^ HOS cells, early and late RT products followed a similar trend (Figure **4A and C**). However, at 48 and 72 hours, late RT products measured in ρ^+^ HOS and cybrid cells were significantly higher than in ρ^0^ HOS cells (Figure **4A**), which was probably due to different levels of integrated viral DNA in those cells. The progress of reverse transcription was evaluated by comparison of late RT products (2, 6 and 10 hours post-infection) with strong stop DNA (2 hour post-infection) (Figure **4D**). A similar trend was found in all the cell lines. At 2 hours post-infection, approximately 4% strong stop DNA was converted into late RT products. At 6 and 10 hours, late RT products reached the maximum level and about 30% of strong stop DNA was converted into full-length late RT products (Figure **4D**). These results indicate that the ability to support reverse transcription was the same in HOS, cybrid and ρ^0^ cells. Therefore, the poor infection of ρ^0^ cells by HIV-1 was due to the inhibition of steps that occur after reverse transcription takes place.

**Figure 5:**
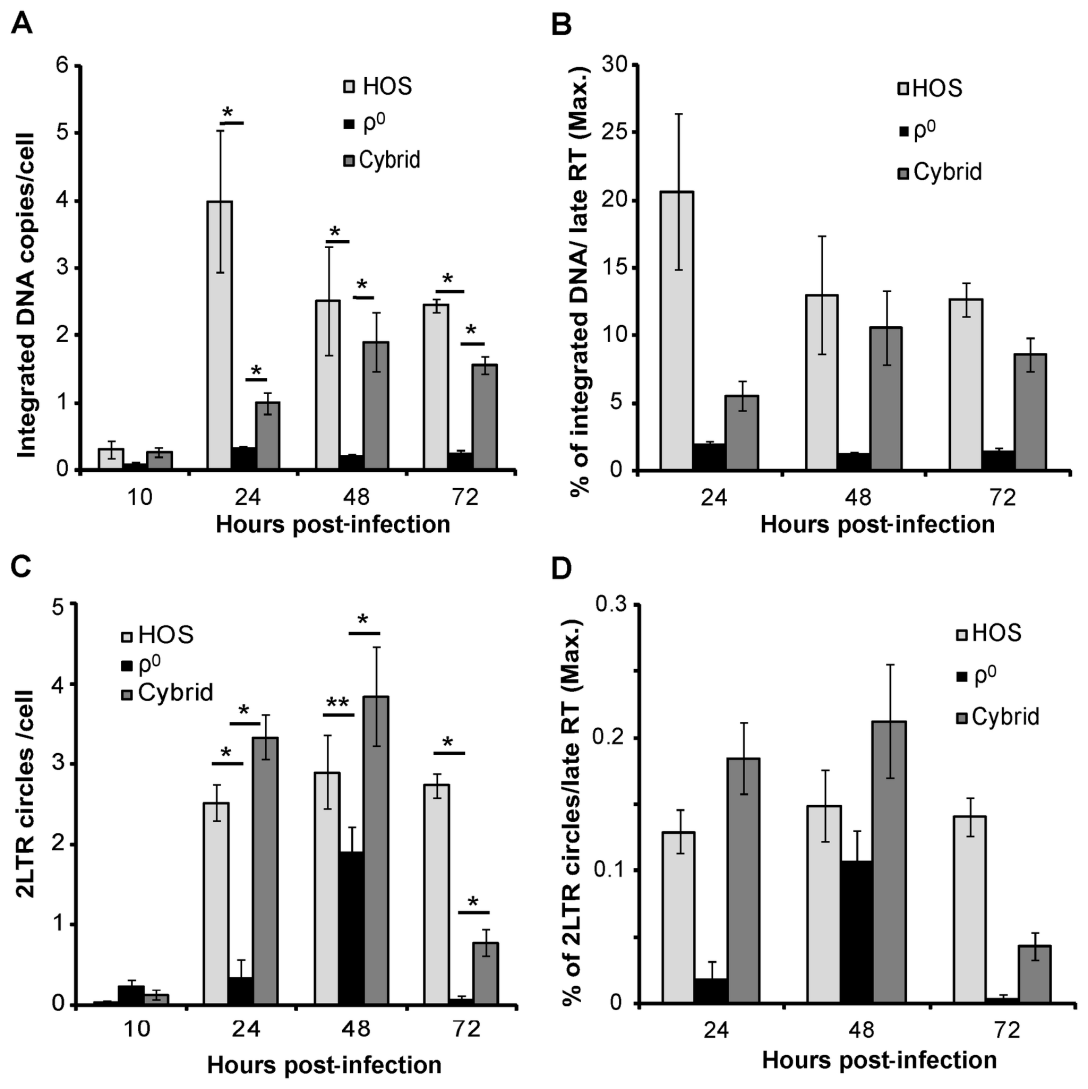
Levels of 2LTR circles and integrated DNA in ρ^0^ cells are significantly lower than in HOS and cybrid cells. (*A*) Levels of integrated DNA detected by Alu-LTR based real-time nested-PCR. (*B*) Integration efficiency shown by the comparison of integrated DNA with late RT product. (*C*) 2-LTR circles detected by real-time PCR. (*D*) Nuclear import efficiency evaluated by the comparison of 2LTR with late RT product (maximum product); HIV-GFP cDNA values are shown per cell (RNase P). Error bars indicate standard deviation of triplicate values. Data are representative of three independent experiments. * *P*<0.01; ** P<0.05.

Subsequently, integrated HIV-1 DNA was quantified by the Alu-LTR based real-time nested-PCR procedure[36]. In contrast to the similar levels of late RT product in all cell lines, integrated HIV-1 DNA in ρ^0^ cells was significantly lower than in HOS and cybrid cells (Figure **5A**). At 72 hours post-infection, the integrated HIV-1 DNA level in ρ^0^ cells was 9-fold lower when compared with HOS cells and 6-fold lower when compared with cybrid cells (Figure **5A**). The percentage of integrated DNA in the total late RT products (maximum level) in HOS, cybrid and ρ^0^ cells were 12.7%, 8.6% and 1.5% respectively (Figure **5B**). This result suggests that infection of ρ^0^ cells by HIV-GFP was blocked either during integration or the steps prior to integration. 

 HIV-1 2LTR circles are the products of non-homologous end-joining DNA repair events and are exclusively found in the nucleus, and usually serve as a marker of viral nuclear import in the studies of viral trafficking. The 2-LTR circle primers amplify the linkage region between 5’ and 3’ LTR ends [37]. In cybrid and HOS cells, 2-LTR circles reached the maximum level at 24 hours post-infection and decreased after 72 hours (Figure **5C**). In contrast, 2-LTR circles in ρ^0^ cells peaked at 48 hours post-infection and then rapidly decreased (Figure **5C**). This delayed accumulation of 2-LTR circles suggested that the intracellular transport of HIV-GFP in ρ^0^ cells was slower than in the HOS and cybrid cell lines. Compared with that in the HOS and cybrid cell lines, the ratio between 2-LTR circles and late RT products (maximum level), which reflects the ability of transporting viral DNA from cytoplasm into nucleus, was significantly lower in ρ^0^ cells (Figure **5D**). This result indicates that the lack of mtDNA in ρ^0^ cells affected the early events of HIV-GFP infection, namely at the steps before nuclear import.

### Viral complexes containing p24 colocalize with mitochondria in the infected cells

**Figure 6:**
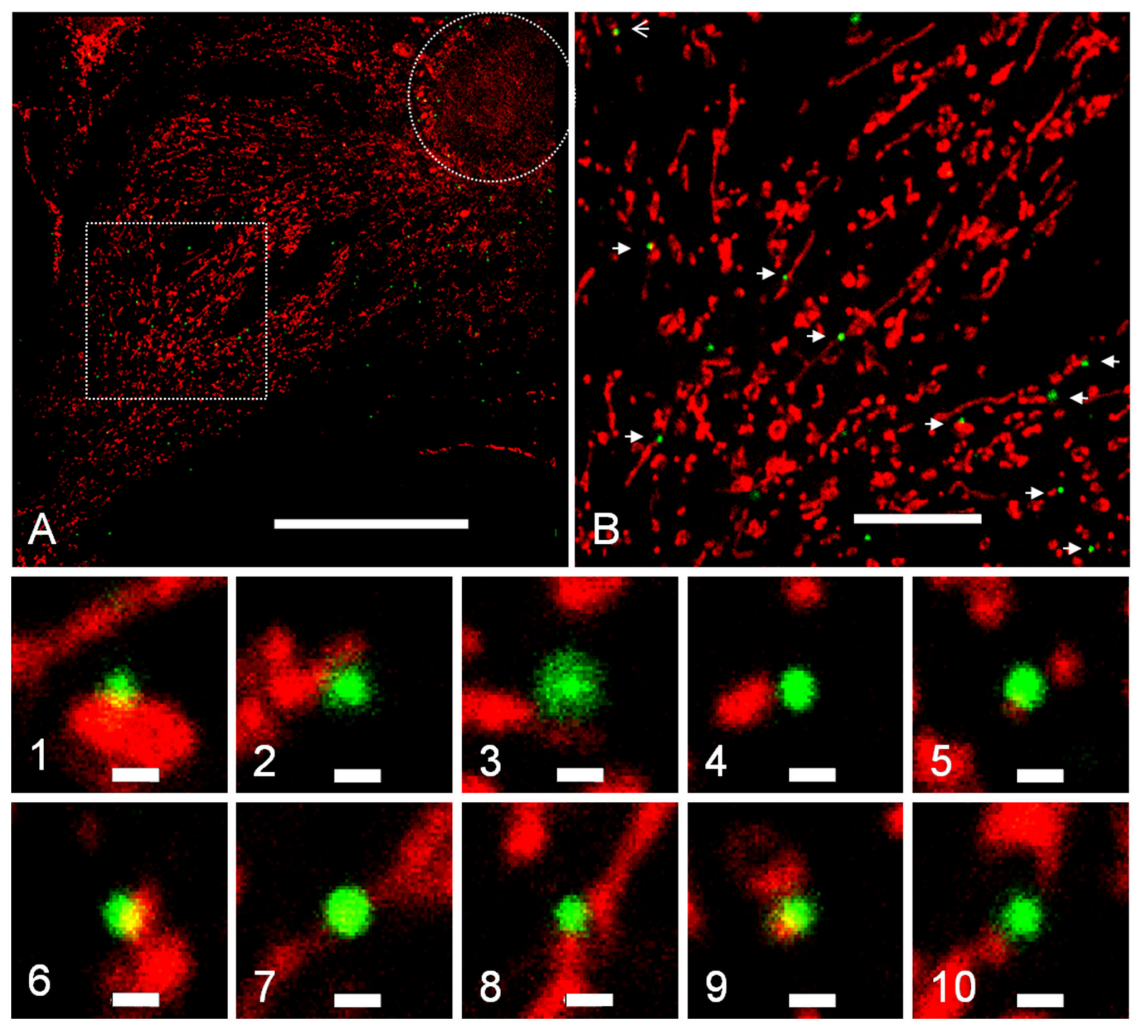
HIV-GFP intracellular complexes co-localize with mitochondria in HOS cells. (*A*) Immunostaining of virus-infected (MOI~10) HOS cells. Mitochondria were labeled with MitoTracker Red (Red) and viral nucleoprotein complexes were detected with p24 antibody and visualized by using Alexa Fluor 488-conjugated second antibody (Green). For orientation, the cell nucleus is marked with a circular dotted white line. (*B*) Enlarged image of the area marked with a square in (*A*) (1-10). Enlarged images of the area marked with white arrow in (*B*) show viral complexes and mitochondria. The scale bars shown in the images are as follows: (*A*) 50 µm, (*B*) 10 µm (1-10), 500 nm.

Deficiency of HIV-GFP infection in ρ^0^ cells suggests that mitochondria might play an important role during the early stages of HIV-1 infection. Further analysis showed that virus infection was blocked at steps after reverse transcription and before nuclear import. We proposed that mitochondria may play a role in the intracellular transport of viral complexes. The localization of viral complexes in the infected cells was determined by immunostaining using confocal microscopy. Six hours post-infection, cells were stained with MitoTracker Red, subsequently fixed with PFA, and viral complexes were detected by a p24 monoclonal antibody (mAb AG3.0) [38-40] and visualized using fluorescently labeled secondary antibody. Figure **6A** shows a representative confocal image from one infected HOS cell. A large fraction of viral complexes containing p24 were found to be associated with, or located in very close proximity to, mitochondria (Figure **6B**, (1-9)). Consistently, a similar result was observed in another cell line (HEK293T) (Figure **S3** in File **S1**). The co-localization of mitochondria with viral complexes indicates that viral complexes may interact with mitochondria during infection. 

## Discussion

Productive and efficient infection by HIV-1 involves numerous cellular host pathways. In this study, we have investigated the contribution of functionally and structurally fit mitochondria to HIV-1 infection efficacy by using a ρ^0^ cell line completely devoid of mtDNA. Our data indicate that ρ^0^ cells are deficient in the ability to support HIV-based lentiviral vector infection and that infection in ρ^0^ cells is blocked at steps that occur after reverse transcription and prior to nuclear import. 

The reason behind the poor infectivity of ρ^0^ cells by HIV-GFP is not directly related to their lack of OXPHOS function since inhibition of OXPHOS by mitochondrial inhibitors does not lead to decreased virus infection in HOS cells. These observations suggest the hypothesis that the OXPHOS system is not important to HIV-1 infection in HOS cells in a functional capacity but rather in a structural capacity, taking into account that ρ^0^ cells have an altered mitochondrial ultrastructure. As an alternative hypothesis, the absence of mtDNA could induce a particular change in gene expression profile that could account for the poor HIV-GFP infection in ρ^0^ cells. Although studies in yeast have shown that antimycin A-treated yeast cells have a similar gene transcript profile to yeast ρ^0^ cells [41], a similar genomic study with the human cells used here could provide additional information for the determination of the role of mitochondria in HIV-1 infection.

Our results with HIV-GFP contrast with the requirement of mitochondrial OXPHOS function for infection by rubella virus (RV) [42], although RV is single-stranded RNA virus that replicates in the cytoplasm. In agreement with our results, it was previously reported that ρ^0^ HOS cells infected by RV yield virus titers significantly lower than the parental ρ^+^ HOS cell line [42]. In contrast, however, when ρ^+^ HOS cells pretreated with respiratory chain inhibitors (antimycin A) or cultivated under (mild) hypoxic conditions to repress mitochondrial metabolism were infected with RV, viral replication was reduced in a time-dependent fashion [42]. The reason behind this discrepancy most probably lies in the difference in the life cycle of the two viruses, because RV induces cluster formation of mitochondria and it is believed that the mitochondrial protein p32 fulfills an essential function for RV replication in directing microtubule-dependent trafficking of mitochondria near sites of viral replication to meet the energy demands of the virus [42]. Similar mitochondria migration patterns have been described for African swine fever virus-infected [43] and hepatitis B virus-infected cells [44]. However, there are several examples of successful establishment of viral infections that do not require mitochondrial OXPHOS. For example, treatment of Rous sarcoma virus-infected cells with chloramphenicol, a mitochondrial protein synthesis inhibitor, or ethidium bromide, which interferes with mtDNA replication, impaired mitochondrial functions, but not virus production or viral RNA synthesis [45]. Hence, a subset of viruses exploit host cell mitochondria and OXPHOS function for productive infection. Among them, HIV-1 infection involves a mechanism independent of host cell OXPHOS.

The HIV-GFP infection in ρ^0^ cells is blocked at steps that occur after reverse transcription and prior to nuclear import. This observation could suggest the possibility that nuclear viral import is prevented in ρ^0^ cells. A more attractive alternative hypothesis would involve a role for mitochondria in viral complex intracellular transport. Although the intracellular behavior of HIV-1 has been studied in great detail [39], a role for mitochondria in the early stages of HIV-1 infection has not previously been reported. Fluorescence-based studies have suggested that HIV-1 intracellular complexes associate with microtubules and use cytoplasmic dynein and the microtubule network to migrate toward the nucleus [39]. Here, we have shown a large fraction of virus complexes containing p24 capsid proteins (pre-integration complexes or PICs) to be near or in contact with mitochondria. However, our data is not conclusive as the interaction could be indirect. For example, since mitochondria and the cytoskeleton are functionally and structurally interconnected [46], the co-localization of PICs with mitochondria may represent the indirect result of the interaction of HIV complexes with microtubules or actin microfilaments [47]. As a speculation, the altered mitochondrial structure in ρ^0^ cells could prevent, either directly or indirectly, proper viral migration via the microtubular network. Alternatively, the PICs could interact independently with both the mitochondria and the cytoskeleton, which could determine their fate. For example, by associating with mitochondria, viral complexes may escape from the cellular degradation machinery and successfully reach the nuclear membrane.

It has been reported that the nucleocapsids of several viruses, including the vesicular stomatitis virus, may associate with or be in close proximity to mitochondria [48]. Rubella virus capsid has been shown to associate with mitochondria by interacting with gC1qR/p32 (mainly located in mitochondria), an association that is important for rubella virus infection [18,42]. Future work should be dedicated to exploring whether HIV-1 intracellular complexes associate with mitochondria and if so, what is the functional significance of this interaction. However, it must be taken into account that for our studies we have used GFP-encoding reporter virus pseudotyped with vesicular stomatitis virus (VSV-G) envelope glycoprotein. This is a widely-used model for analysis of early stage of HIV infection [49-52] since it provides relatively high level of viral entry and detectable amount of PICs due to the 20- to 130-fold higher infectivity than the virus containing HIV envelope [53,54]. However, VSV-G pseudotyped HIV is not identical to the genuine HIV particles. Genuine HIV enters the target cell through a pH-independent membrane fusion process mediated by the interaction between HIV-1 envelope glycoprotein and cell surface receptor CD4 and co-receptors CCR5 or CXCR4 [55,56]. Those cellular receptors are chemokine receptors, which are involved in intracellular signal transduction. It has been reported that interaction between HIV-1 and CXCR4 causes the activation of its downstream signals leading to depolymerization of F-actin, which is necessary for HIV entry process [57]. Differently, the presence of an VSV-G envelope determines the endocytic pathway of virus entry rather than fusion with the plasma membrane. This pathway is known to change subsequent events of PIC maturation and transport, particularly capsid uncoating and nuclear import [58]. In this context, the observed interaction of PICs (specifically p24 capsid protein, i.e. HIV-1 intracytoplasmic complexes with uncoated capsid) with mitochondria may be specific for VSV-G dependent pathway. To address this point future work should be devoted to replicate the present study using cells infected with, for example, murine leukemia virus (MLV) Env-pseudotyped virus, an envelop that determines the entry mechanism similar to HIV Env.

 In conclusion, the results presented in this manuscript indicate that mitochondria play an OXPHOS-independent key role during the early stages of HIV-1 infection. Further studies in this line may lead to the identification of new targets for the development of anti-HIV-1/AIDS therapies.

## Materials and Methods

### Ethics Statement

N/A.

### Cell lines and antibodies

HEK293T cells and HOS cell line (HOS-CD4-CCR5) (obtained from the NIH AIDS Research and Reference Reagent Program) were grown in Dulbecco’s modified Eagle’s medium (DMEM) supplemented with 2 mM glutamine, 10% fetal calf serum and 50 µg/ml gentamicin (complete medium). ρ^0^ cells (143B206) were a derivative of the 143B.TK^-^ osteosarcoma cell line [21] and were originally obtained from its creator Dr. Giuseppe Attardi. ρ^0^ cells were grown in complete DMEM medium supplemented with 10mM sodium pyruvate and 50 µg/ml uridine. All cell lines were cultured at 37°C in 5% CO_2_ (standard condition). Mouse anti-p24 monoclonal antibody (mAb AG3.0) was obtained from the NIH AIDS Reagent Program. Alexa-488 conjugated anti-IgG antibody and MitoTracker Red were purchased from Invitrogen. Transfer plasmid pWPI was purchased from Addgene (plasmid 12254, Didier Trono’s lab). 

### Virus production

VSV-G-pseudotyped HIV-1 vectors (HIV-GFP) were made in HEK293T cells. Briefly, the cells (1X 10^7^) were seeded in 75 cm^2^ flasks and transfected the following day using Polyfect transfection reagent (Qiagen) with 4 µg of plasmid pWPI (GFP), 4 µg of plasmid pCMV-dR8.91 (gag-pol), and 4 µg of plasmid pMD2.G (VSV-G) per flask. After culturing for 24 hours, cells were washed extensively, and complete medium were added. The virus-containing supernatant was collected between 48 hours and 96 hours after transfection and filtered through a 0.22-µm-pore-size filter (Millipore). The filtered supernatant was concentrated using centrifugal filter units (Ultracel-100K) (Millipore), and aliquots were frozen and stored at −140°C. The capsid (p24) contents of the virus stocks were quantified using Alliance ® HIV-1 p24 ELISA Kit (Perkin Elmer) following the manufacturer’s instructions.

### Virus infection in HOS and ρ^0^ cells

HOS cells or ρ^0^cells (2.5X10^4^/well) were seeded in 24-well plates in complete DMEM supplemented with 50 µg/ml uridine and 1 mM sodium pyruvate and incubated overnight at 37°C. The next day, HIV-GFP was treated with 200U/ml DNase I (RNase free) (NEB) for 2 hours at 37°C. Different amounts of DNase I-treated HIV-GFP (2, 5, 10, 20 ng of p24) were added to the cells in a total volume of 0.5ml/well. Two days after infection, the cells were detached using 0.025% trypsin and subsequently fixed in 1% paraformaldehyde (PFA). Infection efficiency was assessed by measuring green fluorescent protein (GFP) expression using cytofluorometry (FACSCalibur; BD Biosciences). A minimum of 10000 events was acquired for each sample, and results were analyzed using Flow Jo 7.6 (Tree Star, Inc). 

### Viral infection, DNA isolation and probe-based real-time quantitative PCR (TaqMan)

HOS, ρ^0^ or cybrid cells (2 X 10^5^/well in 6-well plates) were seeded in complete DMEM supplemented with 50 µg/ml uridine and 1 mM sodium pyruvate and incubated overnight at 37°C. The next day, HIV-GFP was treated with 200 U/ml DNase I for 2 hours at 37°C before adding to the cells. Cells were then infected with an equivalent of 20 ng of p24 of HIV-GFP (DNase I-treated) per well in 1.5ml medium in the presence of 10 µg/ml polybrene and incubated at 37°C. After 2 hours, cells were washed with DMEM and fresh medium was added. Cells were collected at 2, 6, 10, 24, 48 and 72 hours post-infection and washed 3 times with PBS, and total cellular DNA was harvested using the DNeasy Blood &Tissue Kit (Qiagen, N.V.) according to the manufacturer’s instructions. Different forms of viral cDNA were quantified by real-time PCR using published primers or primers designed specifically for our HIV-GFP genome sequences as explained in the supplementary material.

### Statistical analyses

All experiments were done at least in triplicate. All data are presented as means ± SD of absolute values or percent of control. Values were analyzed for statistical significance by Student’s t-test. P < 0.05 was considered significant.

## Supporting Information

File S1 **This includes 3 Figures, 1 Table and Supplementary Materials and Methods.**
(PDF)
