# Supplementary material for: HIV-1 Infection Is Blocked at an Early Stage in Cells Devoid of Mitochondrial DNA
Source: PLoS One. 2013 Oct 21;8(10):e78035. doi: 10.1371/journal.pone.0078035 (PMC3804459; doi:10.1371/journal.pone.0078035)
Supplement: File S1 — This includes 3 Figures, 1 Table and Supplementary Materials and Methods. (PDF) [file pone.0078035.s001.pdf]

Supplementary material to:

HIV-1 Infection is Blocked at an Early Stage in Cells Devoid of Mitochondrial DNA

Gaofei Lu[1], Suzanne E. Matsuura[1], Antoni Barrientos[1,2*] and Walter A. Scott[1#]
[1]*Department of Biochemistry and Molecular Biology,* [2]*Department of Neurology. University of Miami Miller School of Medicine. Miami, Florida, USA.*

Inventory

- Supplementary Materials and Methods
- Supplementary Tables
- Supplementary References
- Supplementary Figures and Figure Legends

Supplementary Materials and Methods

Probe-based real-time quantitative PCR (TaqMan). Quantitative real-time PCR was performed in triplicate, in SsoFast Probes Supermix (Bio-Rad, Hercules, CA) using final concentrations of 250 nM each for the forward and reverse oligonucleotide primers, and 200 nM for the probes. Thermal-cycling conditions were 3 min at 95°C, 15 s at 95°C and 1 min at 60°C for 40 cycles using the Bio-Rad CFX96 real-time PCR detection system. Results were analyzed with CFX Manager (Bio-Rad). Primer sequences and probes used in this study were shown in **Table S1**. Late RT primers (MH531 and MH532) [1] amplify the region that flanks the primer binding site (PBS) and will only detect full-length viral DNA. In contrast, primer pair for total RT products (MH535 and early reverse 2) is specific for the R/U5 region of the viral long terminal repeat (LTR) and detects virtually all HIV-1 DNA synthesized including early RT products (strong stop DNA), late RT products (full length cDNA) and partial RT products. Linearized pWPI plasmids were used as the standard in the measurement of total and late RT products. Integrated HIV-1 DNA was measured by Alu-LTR based real-time nested-PCR procedure [2]. Total cellular DNA isolated from infected HOS cells 40 days post-infection (17% GFP-positive) was used as standard. Integrated HIV-1 DNA in the standard was quantified using the late RT primer set. DNA fragments containing 2LTR junction sequence (U5/U3) or mtDNA (nucleotide position 10620-10710) were cloned into plasmid containing pGEX-3X (Addgene) backbone separately and the recombinant plasmids (p2LTR or pGST-MT) were used as standards in the measurement of 2LTR circles or mtDNA. Cell number variation was measured using real-time PCR for RNase P DNA by using Taqman Copy Number Reference Assay, RNase P (Invitrogen). Cellular DNA isolated from a known

number of HOS cells was used as standard in the measurement of cell number variation.

Effect of mitochondrial inhibitor on virus infection. HOS cells were seeded in 12-well plates at a concentration of 4 X 10^4/well and incubated overnight at 37°C. Cells were pre-incubated with various concentrations of different mitochondrial inhibitors (5 μg/ml antimycin A, 10 μg/ml oligomycin and 10 μM carbonyl cyanide 3-chlorophenylhydrazone (CCCP) (Sigma) for 6 hours with or without the supplementation of 1 mM sodium pyruvate and 50 μg/ml uridine, and cells were subsequently infected with an equivalent of 10 ng of p24 of HIV-GFP. Two hours after infection, the medium was replaced with fresh medium containing different mitochondrial inhibitors. Infection was assessed 2 days later by FACS analysis as described in the main text.

Repopulation of ρ^0 cells with functional mtDNA. Reintroduction of mtDNA from HEK293T cells into ρ^0 HOS 143B-TK^- cells was performed essentially as described [3,4]. The use of a TK^+ cell line, such as HEK293T, as mtDNA donor was required to ensure a proper cybrid selection. HEK 293T cells were seeded in 6-well plates and grown to 70~80% confluency. Cells were enucleated by the treatment with 3 μg/ml actinomycin D for 20 hours and washed with DMEM for 5 min to remove the drug. ρ^0 cells (1X10^6/well) were added on top of actinomycin D treated HEK293T cells and incubated at 37 °C for 5 hours allowing ρ^0 cells to attach and make contact with HEK293T cells. Cells were washed three times with DMEM without FBS. One ml of freshly prepared fusion medium (1 g/ml PEG 1450, 20% DMSO in DMEM) was added to the cells and incubated for 2 min at room temperature. The cells were washed with DMEM three times

and incubated overnight in complete medium. The cells were detached and reseeded at low concentration (1:20 dilution) in 6-well plates in DMEM supplemented with 10% dialyzed FBS and 100 μg/ml bromodeoxyurine (BrdU), to select against expression of the TK^+ phenotype of the HEK293T cells, and in the absence of uridine and pyruvate, to select against the respiratory-null unfused ρ^0 cells [3]. Medium was changed every 3 days. Therefore, only the ρ^0 cells that had fused with cytoplasts could survive in this medium, while the ρ^0 cells that had not fused or that had fused with residual intact HEK293T cells, as well as any residual intact HEK293T cells, were eliminated. Colonies were visible after 3 weeks of incubation at 37°C. Single clones were isolated using cloning rings and trypsin treatment. Isolated cybrid cell clones were grown in complete DMEM in the absence of pyruvate and uridine.

Confocal microscopy for immunofluorescence analysis. Cells (HOS or HEK293T) were grown on glass coverslips in 6-well plates and infected with HIV-GFP. Six hours post-infection, 300 nM MitoTracker Red (Invitrogen) was added to the cells and incubated for another 30 min. Cells were washed 3 times using DMEM and once with PBS. Samples were fixed with 3.75% paraformaldehyde (PFA) in PBS for 20 min at room temperature and subsequently washed 5 times with PBS. Fixed cells were permeabilized with 0.0025% digitonin in PBS for 20 min at room temperature and subsequently blocked with blocking buffer (3% BSA, 0.0025% digitonin in PBS). Samples were incubated with mouse anti-p24 monoclonal antibody (diluted 1:100 in wash buffer) overnight at 4°C in a humidified atmosphere followed by incubation with Alexa 488-conjugated anti-mouse IgG secondary antibody (1:300 in wash buffer) for 2

hours at room temperature. Coverslips were then mounted onto glass slides with Prolong Gold Antifade reagent (Invitrogen). Imaging was performed with a Leica SP5 spectral confocal inverted microscope (SP5 inverted) or a laser-scanning confocal microscope (LSM510, Carl Zeiss). Cell images were acquired by using the 63X oil objective. Optical slices were taken at 200nm intervals along the z-axis through the entire depth of the cell layer. Appropriate laser lines for each fluorophore were used: 488nm line for Alexa Fluor 488 and 543-nm line for MitoTracker Red. Images were analyzed using ImageJ, Zeiss LSM Image Browser and LAS AF Lite software.

Supplementary Tables

Table S1. Primers and probes used in this study.

Target DNA	Primer or probe	Name and sequence	Ref.
Total RT (R/U5)	Early RT Forward	MH535: 5’-AACTAGGGAACCCACTGCTTAAG-3’	[1]
	Early RT Reverse	Early2: 5’-CTGCTAGAGATTTTCCACAC-3’	TS
	Early RT probe	MH603: 5'-(FAM)-ACACTACTTGAAGCACTCAAGGCAAGCTTT-(BHQ)-3'	[1]
Late RT (flank PBS)	Late RT forward	MH531: 5’-TGTGTGCCCGTCTGTTGTGT-3’	[1]
	Late RT reverse	MH532: 5’-GAGTCCTGCGTCGAGAGAGC-3’	[1]
	Late RT probe	LRT-P: 5’-(FAM)-CAGTGGCGCCCGAACAGGGA-(BHQ)-3’	[1]
Integrated DNA (Alu-LTR)	First round Alu-LTR PCR		
	LTR forward	GSTLTR: 5‘-AGACAGATAGGGCCGTTAAACTAGGGAACCCACTGCTTAAG-3’	TS
	Alu reverse	Alu2: 5’-GCCTCCCAAAGTGCTGGGATTACAG-3’	[2]
	Second round real time PCR		
	GST forward	GST: 5’-AGACAGATAGGGCCGTTAAAC-3’	TS
	LTR reverse	Early2: 5’-CTGCTAGAGATTTTCCACAC-3’	TS
	Probe	MH603: 5'-(FAM)-ACACTACTTGAAGCACTCAAGGCAAGCTTT-(BHQ)-3'	[1]
2LTR Circle (U5/U3)	2LTR forward	MH535 : 5’-AACTAGGGAACCCACTGCTTAAG-3’	[1]
	2LTR reverse	2LTR-3: 5’-GTTATTCCCATGGTGAATTGATCC-3’	TS
	Probe	MH603: 5'-(FAM)-ACACTACTTGAAGCACTCAAGGCAAGCTTT-(BHQ)-3'	[1]
mtDNA (10620-10710)	mtDNA forward	MH533: 5'-ACCCACTCCCTCTTAGCCAATATT-3’	[1]
	mtDNA reverse	MH534: 5'-GTAGGGCTAGGCCCACCG-3’	[1]
	mtDNA probe	mito-probe: 5’-(FAM)-CTAGTCTTTGCCGCCTGCGAAGCA-(BHQ)-3’	[1]

TS: This Study

Supplementary References

1. Butler SL, Hansen MS, Bushman FD (2001) A quantitative assay for HIV DNA integration in vivo. Nat Med 7: 631-634.
2. Brussel A, Sonigo P (2003) Analysis of early human immunodeficiency virus type 1 DNA synthesis by use of a new sensitive assay for quantifying integrated provirus. J Virol 77: 10119-10124.
3. King MP, Attardi G (1989) Human cells lacking mtDNA: repopulation with exogenous mitochondria by complementation. Science 246: 500-503.
4. Moraes CT, Dey R, Barrientos A (2001) Transmitochondrial technology in animal cells. Methods Cell Biol: 397-412.

Supplementary Figures and Figure Legends

Figure S1

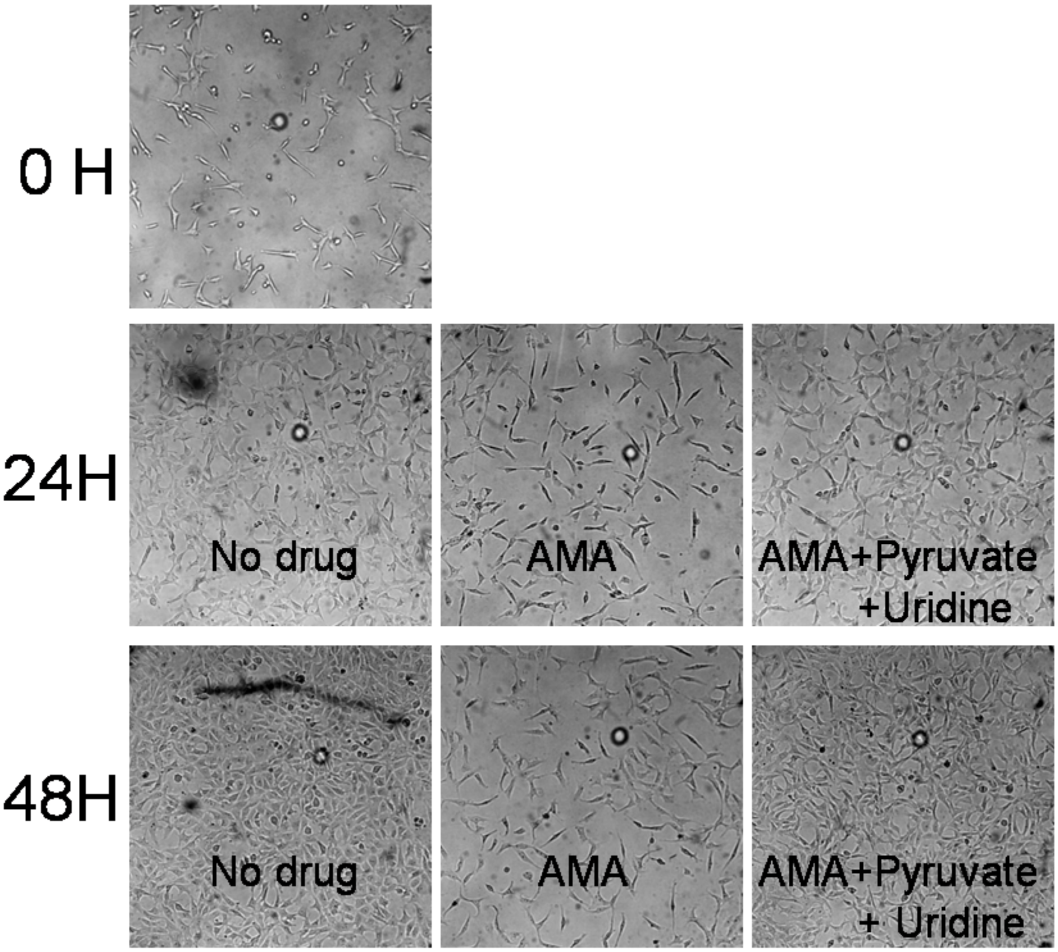

Fig S1. Effect of Antimycin A (AMA) in cellular growth. HOS cells were grown in 12-well plates overnight. The next day, cells were treated with AMA with or without the supplementation of pyruvate and uridine. Cells images were taken at different times (0, 24h and 48h) after the drug treatment indicated in the figure.

Figure S2

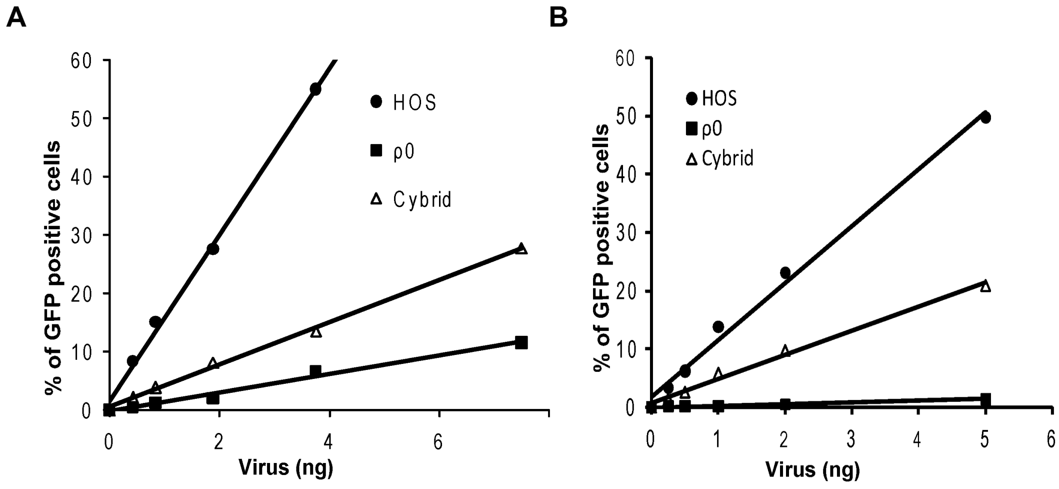

Fig. S2. Infection efficiency by HIV-GFP is significantly restored in a transmitochondrial cybrid line. Estimation of cellular infectivity by HIV-1 in HOS, cybrid and ρ^0 cells. HOS, cybrid and ρ^0 cells were infected with HIV-GFP virus in the presence of 10 µg/ml polybrene for 5 hours and the medium was subsequently replaced with fresh complete medium. Two days later the percentage of GFP expression was measured by FACS analysis and was plotted against the amount of virus used in each experiment. (*A*) and (*B*) represent the data obtained in two independent experiments. In each case, the intra-experimental variability was near zero.

Figure S3

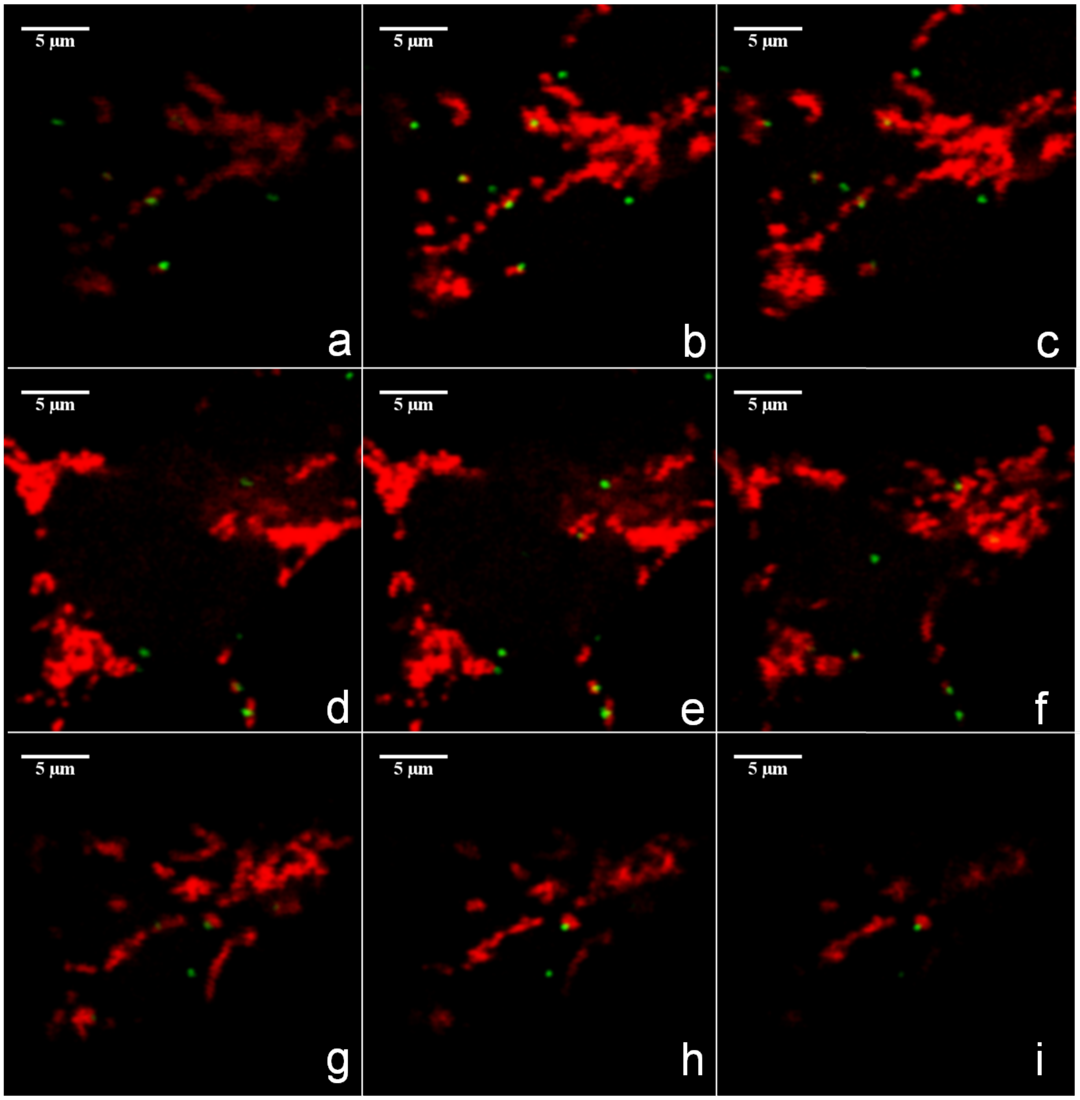

Fig. S3. Co-localization of HIV intracellular complexes with mitochondria in HEK293T cells. HEK293T cells seeded on the coverslip were infected with virus (MOI~5) for 6 hours. Mitochondria were labeled with MitoTracker red (Red). Viral complexes were detected with a monoclonal anti-p24 antibody and visualized with Alexa 488-conjugated anti-mouse IgG secondary antibody (Green). Imaging was performed with a laser-scanning confocal microscope (LSM510, Carl Zeiss). Cell images were acquired by using the 63X oil objective lens, with zoom factor of 2. Optical slices were taken every 200nm interval along the z-axis covering the whole depth of the cell. Images were analyzed using ImageJ and Zeiss LSM Image Browser. Images (*A-I*) are representatives of a series of images of one representative infected cell captured at different focal depths along the z-axis.
